# Microneedling-induced sarcoidal granulomatous reaction

**DOI:** 10.1016/j.jdcr.2026.03.046

**Published:** 2026-03-27

**Authors:** Cemre B. Turk, Afsaneh Alavi, Katelyn R. Anderson, Carilyn N. Wieland, Reese L. Imhof

**Affiliations:** Department of Dermatology, Mayo Clinic, Rochester, Minnesota

**Keywords:** cosmetics, granulomatous dermatoses, microneedling, sarcoidal granulomatous reaction

## Introduction

Microneedling is a popular, minimally invasive cosmetic procedure that promotes dermal remodeling through controlled microtrauma and is widely used for skin rejuvenation and scar revision. Although generally safe, adverse reactions such as infection, scarring, and granulomatous reactions have been reported.^1^^,^^2^ Cutaneous sarcoidal granulomatous reactions may mimic or unmask systemic sarcoidosis and pose significant diagnostic and therapeutic challenges.^3^, ^4^, ^5^ Prior reports have primarily described cases triggered by topical agents applied during microneedling, such as vitamin C serums, and treated with corticosteroids.^3^^,^^6^

We present a case of microneedling-induced cutaneous sarcoidal granulomatous reaction.

## Case report

A 79-year-old woman presented with a 4-month history of a painful rash on the face and upper chest that developed following a microneedling procedure performed at a medspa. She described the eruption as beginning approximately 3 days after the microneedling procedure, initially with “small pink bumps” that progressively became “red, blistered, and peeling,” accompanied by marked pain and tenderness. She denied systemic symptoms aside from intermittent headaches. She had a prior history of venous thrombosis managed with aspirin and was otherwise without significant medical comorbidities.

The microneedling procedure was performed on both the face and upper chest. The patient denied application of any over-the-counter, compounded, or unregulated topical products before or after the procedure, to the best of her recollection. She was initially managed for presumed inflammatory or contact dermatitis and treated sequentially with low-potency, medium-potency, and high-potency topical corticosteroids without clinical improvement. During this period, the eruption remained severely painful and progressive, and topical corticosteroids were continued without benefit. A brief course of systemic corticosteroids (prednisone 40 mg daily for 4 days, 0.75 mg/kg) was subsequently trialed, also without improvement. She was then referred to a local dermatologist, who performed a skin biopsy from the upper chest demonstrating sarcoidal granulomatous inflammation. Lesions on the face and chest were morphologically similar. Given persistent, progressive disease and biopsy findings, methotrexate 15 mg weekly was initiated but discontinued after approximately 4 weeks due to lack of response and patient-reported headaches, with the eruption remaining severely painful and progressive.

Approximately 1 month later, she independently developed an episode of lower-extremity cellulitis and was prescribed oral doxycycline 100 mg twice daily by an urgent-care provider. Within approximately 2 weeks of initiating therapy, she noted mild improvement with decreased tenderness and reduced erythema of the papules and subsequently presented to our clinic for further evaluation.

**Fig 1:**
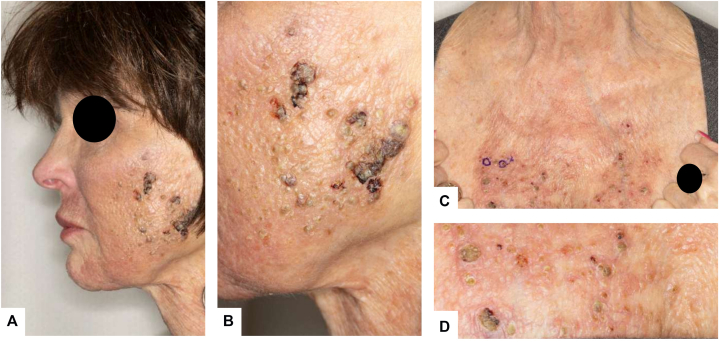
Skin findings before treatment. **(A)** Face and neck showing erythematous perifollicular and nonfollicular papules with a yellowish hue and focal crusting. **(B)** Close-up of the malar cheek with coalescing papules, pustules, and serohemorrhagic crust. **(C)** Upper chest with scattered erythematous papules exhibiting an orange-yellow hue. **(D)** Close-up of the upper chest showing shiny erythematous-yellow papules.

**Fig 2:**
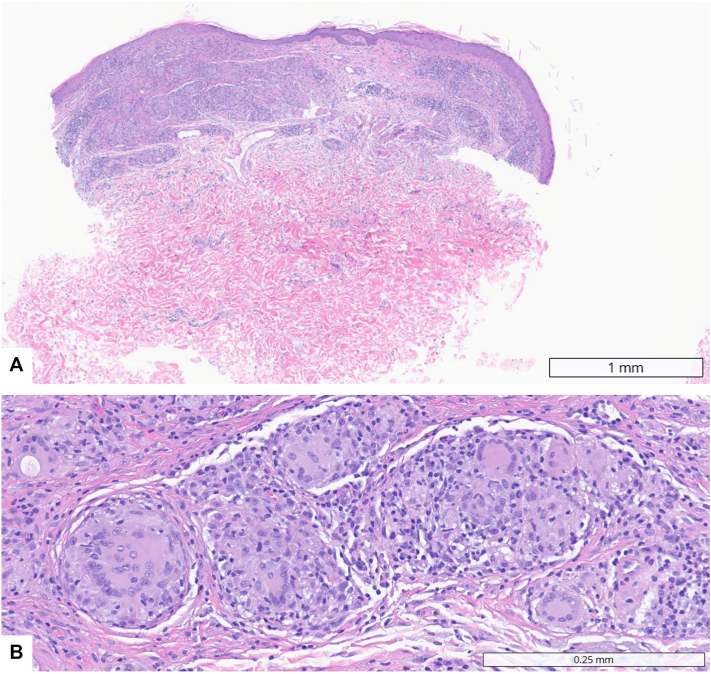
Histopathologic findings of microneedling-induced granulomatous inflammation. **(A)** Low-power view showing dermal noncaseating granulomatous inflammation extending into the upper dermis (H&E; scale bar = 1 mm). **(B)** High-power view demonstrating compact sarcoidal granulomas composed of epithelioid histiocytes and multinucleated giant cells with sparse lymphocytic infiltrate (H&E; scale bar = 0.25 mm).

On examination, numerous 1-3 mm erythematous perifollicular and nonfollicular papules with a yellowish hue and pustules, several demonstrating superficial erosion with overlying serohemorrhagic crust, were distributed across the face and upper chest on a background of diffuse erythema (Fig 1). Two punch biopsies from the upper chest revealed dermal noncaseating granulomatous inflammation composed of epithelioid histiocytes and multinucleated giant cells with surrounding lymphocytic infiltrate (Fig 2). Grocott-Gomori methenamine silver, acid-fast bacillus, and Fite stains, as well as tissue cultures, were negative for microorganisms. An extensive laboratory and imaging evaluation excluded systemic sarcoidosis. The patient was diagnosed with a microneedling-associated cutaneous sarcoidal granulomatous reaction.

**Fig 3:**
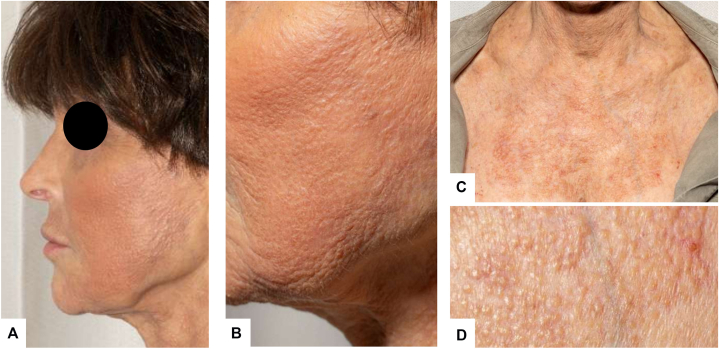
Clinical improvement after treatment. **(A)** Face and neck showing marked improvement. **(B)** Close-up of the malar cheek demonstrating resolution of pustules and crusting with significant reduction in papules. **(C)** Upper chest with faint residual erythema and scattered papules. **(D)** Close-up of the upper chest showing near-complete resolution of lesions after 3 months of doxycycline and topical tacrolimus therapy.

She was treated with oral doxycycline 100 mg twice daily and topical tacrolimus 0.1% ointment twice daily, resulting in marked improvement and significant reduction of lesions over a 3-month period (Fig 3).

## Discussion

Microneedling is widely regarded as a safe and effective technique for skin rejuvenation and scar revision; however, granulomatous reactions have been increasingly recognized as rare adverse events.^1^, ^2^, ^3^ These reactions are most frequently associated with motorized microneedling devices and the concurrent application of topical agents (including vitamin C serums, hydrating gels, and pigment stabilizers) which facilitate dermal penetration of immunogenic material and may elicit a delayed-type hypersensitivity response.^5^^,^^6^ Granulomatous hypersensitivity reactions have been associated with intradermal introduction of various particulate or ionized materials, including metallic elements, zirconium, silica, and components of cosmeceutical formulations such as preservatives or unlisted ingredients.^6^ The proposed pathogenesis involves a combination of local immune dysregulation within previously traumatized or scarred skin, an “immunocompromised district”, with subsequent antigen-driven granuloma formation, similar to mechanisms seen in cutaneous sarcoidosis and other granulomatous disorders.^4^^,^^5^^,^^7^

Clinically, microneedling-induced granulomatous reactions may mimic or unmask cutaneous sarcoidosis, posing both diagnostic and therapeutic challenges. Histopathologic examination typically reveals noncaseating epithelioid granulomas, necessitating careful exclusion of infectious etiologies and consideration of alternative granulomatous processes, including foreign body–type reactions related to exogenous material introduced during microneedling, delayed-type hypersensitivity reactions to topical products, granulomatous rosacea, Crohn’s-associated granulomatous dermatitis, and other inflammatory granulomatous dermatoses such as granulomatous periorificial dermatitis.^6^ Infectious granulomatous processes, including atypical mycobacterial and deep fungal infections, must be excluded with appropriate special stains and tissue cultures. Although most postmicroneedling reactions are transient and self-limited, persistent granulomatous inflammation can be refractory to conventional treatment with topical or systemic corticosteroids.^3^^,^^6^ In such refractory cases, second-line systemic agents, including methotrexate or antimalarials, may be employed, although incomplete responses and adverse effects are frequent.^8^

Emerging evidence suggests that tetracyclines (eg, doxycycline, minocycline) may provide benefit in steroid-resistant granulomatous dermatoses due to their immunomodulatory activity and favorable safety profile.^6^^,^^8^ These agents inhibit matrix metalloproteinases, attenuate neutrophil chemotaxis, and suppress proinflammatory cytokines, thereby modulating granuloma formation. Additional options include topical calcineurin inhibitors (such as tacrolimus), tumor necrosis factor α inhibitors, and, in selected cases, isotretinoin or thalidomide.^5^^,^^8^ Given the paucity of robust clinical data, management should remain individualized, balancing efficacy, tolerability, and patient comorbidities.

It is important to note that interpretation is limited by the retrospective nature of the report and reliance on outside documentation; methotrexate and corticosteroid therapy may not have been administered for sufficient duration to achieve maximal benefit, yet clinical improvement was achieved without prolonged systemic immunosuppression. This case highlights an uncommon immune-mediated complication of an increasingly common cosmetic procedure and underscores the potential role of tetracycline-based, steroid-sparing therapy in refractory microneedling-associated granulomatous reactions. As cosmetic dermatology continues to expand beyond traditional medical settings, heightened awareness of such reactions and thoughtful clinician–patient partnership remains essential to ensure procedural safety and optimal clinical outcomes.

## Conflicts of interest

None disclosed.
